# Migration as a form of workforce attrition: a nine-country study of pharmacists

**DOI:** 10.1186/1478-4491-7-32

**Published:** 2009-04-09

**Authors:** Tana Wuliji, Sarah Carter, Ian Bates

**Affiliations:** 1 International Pharmaceutical Federation (FIP), The Hague, The Netherlands; 2 School of Pharmacy, University of London, London, UK

## Abstract

**Background:**

There is a lack of evidence to inform policy development on the reasons why health professionals migrate. Few studies have sought to empirically determine factors influencing the intention to migrate and none have explored the relationship between factors. This paper reports on the first international attempt to investigate the migration intentions of pharmacy students and identify migration factors and their relationships.

**Methods:**

Responses were gathered from 791 final-year pharmacy students from nine countries: Australia, Bangladesh, Croatia, Egypt, Portugal, Nepal, Singapore, Slovenia and Zimbabwe. Data were analysed by means of Principal Components Analysis (PCA) and two-step cluster analysis to determine the relationships between factors influencing migration and the characteristics of subpopulations most likely and least likely to migrate.

**Results:**

Results showed a significant difference in attitudes towards the professional and sociopolitical environment of the home country and perceptions of opportunities abroad between those who have no intention of migrating and those who intend to migrate on a long-term basis. Attitudes of students planning short-term migration were not significantly different from those of students who did not intend to migrate. These attitudes, together with gender, knowledge of other migrant pharmacists and past experiences abroad, are associated with an increased propensity for migration.

**Conclusion:**

Given the influence of the country context and environment on migration intentions, research and policy should frame the issue of migration in the context of the wider human resource agenda, thus viewing migration as one form of attrition and a symptom of other root causes. Remuneration is not an independent stand-alone factor influencing migration intentions and cannot be decoupled from professional development factors. Comprehensive human resource policy development that takes into account the issues of both remuneration and professional development are necessary to encourage retention.

## Background

Migration, a complex phenomenon, has long held centre stage in discussions concerning the human resources for health crisis. The international migration of health professionals is thought to reflect the widening of global inequalities [1]. It is also seen as the cause of deteriorating health systems, working conditions and workforce shortages in developing countries [2-5]. The decimation of the health workforce in developing countries has also been attributed to increasing emigration rates [6]. But recent evidence suggests otherwise, although acknowledges that increasing emigration rates can further exacerbate existing health workforce issues [7,8].

Economic and sociological theories attempting to explain migration dominate the literature, with particular emphasis on "push-pull" factors, labour demand, income differentials and migrant networks [9]. A comprehensive understanding of international migration requires consideration of influences beyond those at the individual and household level, taking into account the influence of the country as a whole and its policies and circumstances, such as the labour market, private and public sectors and sociopolitical contexts [10,11].

While an array of discussion and policy papers, opinion pieces, theoretical explorations and questions has been published, there is little empirical evidence to better understand why skilled workers, particularly health professionals, migrate [12-14]. Postulated reasons for migration arising from studies include better remuneration, joining or supporting family, political and social instability, poor living conditions, poor working conditions and management, unsafe environment, further training and qualifications, and job opportunities and satisfaction [15-21].

The issue of remuneration in source countries is thought to play a significant role and has been identified as a key reason for the international migration of health professionals. From this perspective, source countries are said to be adversely affected by labour market forces with an inevitable "pull" from richer and more-developed countries, thereby depleting human capital (also commonly referred to as "brain drain"). But such perspectives may drive policy development towards a narrow set of interventions without full consideration of the "push" factors and country contexts [10].

Some studies acknowledge reasons for migration beyond remuneration but do not analyse the relationship or associations between the factors influencing migration or develop an understanding of the relative significance of each factor [1,15,19,22,23]. Various studies have investigated the migration intentions of health professionals and students, but few have specifically examined the migration intentions of pharmacy students or pharmacists [1,15,22,24,25].

A recent qualitative study examining the professional aspirations of Ghanaian pharmacy students found that most final-year pharmacy students planned to migrate, with the main reasons for migration cited as further postgraduate study and development of capital for personal development, business and family needs [21]. Students also perceived pharmacists abroad to be better respected and to hold more desirable professional and clinical roles [21]. Interestingly, most of the students interviewed expressed a desire to return to Ghana after achieving their objectives abroad [21].

Over a quarter of Lithuanian pharmacists surveyed in a 2007 study planed to migrate to other European countries, with the main reasons identified as better salaries, quality of life and professional opportunities [20]. Pharmacists with English-language skills were found to be four times more likely to plan to migrate than those without [20].

Pharmacists in both community and hospital settings have been described in the literature as contributing to improved health, reduced morbidity and mortality, prevention of hospital admissions, improved rational use of medicines and increased access to health care and medicines, including underserved populations [26-37]. Evidence supports the extended roles that pharmacists adopt beyond the "traditional" supply of medicines to deliver population-level health promotion services such as health education, HIV and sexually transmitted infection prevention, screening and monitoring for chronic conditions, adherence support for long-term therapies and medicines management services to optimize rational use of medicines.

Despite the significance of pharmacists in the health care system, very little workforce research or policy analysis exists. A recent report of the global body representing pharmacists and pharmaceutical scientists, the International Pharmaceutical Federation (FIP), suggests that there are particularly severe pharmacist workforce shortages and increasing emigration rates in sub-Saharan Africa [38]. According to 2008 pharmacy workforce figures from FIP, there was only one pharmacist for every 140 000 people in Uganda, while there was one for every 1300 in the United Kingdom.

To our knowledge, no study has sought to examine the relationships between the cited factors thought to influence the intention of health professionals to migrate. Without this understanding, categorical lists of independent factors may do little to inform comprehensive policy options for building and strengthening the health workforce where a meaningful package of targeted interventions is required.

This exploratory study does not assume a particular theoretical basis to explain migration, nor does it assume that its findings are generalizable to other health professions. This paper does not present specific country-level analysis or make references to each country's local human resource policy and workforce context, although this is the focus of future research. While the individual served as the basis of analysis, the influence of contextual factors at national and international levels was taken into account.

The purpose of this research was to investigate the migration intentions of final-year pharmacy students and develop greater understanding of the economic, sociopolitical, professional and personal factors that influence the intention to migrate.

## Methods

The definition of migration was adopted from the United Nations and refers to the movement of persons that change their country of usual residence [39]. Final-year pharmacy students were selected as the target group for this study for two reasons. First, pharmacy students were accessible via the International Pharmaceutical Students' Federation (IPSF) network. Second, final-year students were more likely to be certain of their future plans than students in any other year of study. Nine countries were selected for the study, based on the interest of local pharmacy student associations to participate and willingness to gather data, and included Australia, Bangladesh, Croatia, Egypt, Nepal, Singapore, Slovenia, Portugal and Zimbabwe.

The questionnaire tool was developed by the authors, reviewed by experts in the field and revised before being distributed to the international research group (comprising local research teams in each country). Data from each participant country were collated, cleaned and prepared for analysis in SPSS for Windows, version 15. Principal Component Analysis (PCA) and two-step cluster analysis were used to explore the dataset and determine influencing factors of migration intentions.

### Questionnaire development

The dependent variable – the intention to migrate within the next five years – was recorded as no intention, or intentions on a short-term (< 2 years) or long-term basis (> 2 years). This allowed examination of potential differences in the attitudes towards migration between those who did not plan to migrate or planned short-term or long-term migration.

Independent variables were identified, such as gender, country of birth, age, university and country of study. Other variables (unclear causality with the intention to migrate) include knowledge of migrant pharmacists and previous professional experience abroad. The intention to migrate may influence the latter two variables and vice versa. Further exploration of the cause and effect of these variables was not investigated in this study and should be examined via qualitative methods.

The questionnaire also included 20 statements relating to reasons for migration, to which respondents could indicate their response on a five-point Likert scale from 1 (strongly agree) to 5 (strongly disagree). These statements were developed from six thematic constructs that described reasons for migration and included personal status, economics, training and professional development opportunities, cultural issues, politics and perceived professional status. Constructs were identified with a focus group of pharmacy students during the IPSF Congress in Bonn, Germany, in 2005.

### Data collection

The questionnaire was distributed via national research teams to final-year pharmacy students at participating universities. Completed questionnaires were returned to each national research group, which coded and entered the data into a standardized collection spreadsheet in Windows Excel^®^. These were then combined into one centralized dataset. Data were collected over a six-week period in April and May 2006.

A random 4% sample of responses was checked for coding errors. The coding error percentage was negligible at 0.05% across the collated dataset.

### Statistical analyses

Principal Components Analysis (PCA) on the 20 statements yielded three factors. The factors were tested for reliability and inter-item correlation before being coded and used for further analysis. One item was excluded from the factors due to poor loading. The differences in the means between groups and their importance were examined through independent t-tests and calculated effect sizes (*r*).

Two-step cluster analysis was used as an exploratory tool to determine subpopulations or clusters within the dataset. This method enables the input of both categorical and continuous data. The categorical variables included gender, intention to migrate, past pharmacy experience abroad and knowledge of a pharmacist who had migrated. The continuous variables included the three factors derived from PCA (Factors 1, 2 and 3).

### Bias and limitations

Respondents may be self-selected, in that those intending to migrate were possibly more likely to complete the questionnaire. However, given the response rate in most countries, this is likely to be a small effect, though potentially more significant in Portugal and Slovenia, where there was a lower response. Data in Egypt were collected at a student forum rather than at individual universities and hence may not be a representative sample. The response rate for Bangladesh was unknown.

Migration intention studies do not necessarily reflect actual migration, nor are they reliably predictive of future trends, as they are likely to overestimate planned migration [23]. However, this approach sheds light on the extent to which the intention to migrate exists and key issues that are associated with these intentions. Findings can be used to inform the development of workforce and education policy development that encourage retention.

## Results

### Sample

An overall response rate of 75.5% was achieved in the study (Table 1), with a total of 984 questionnaires disseminated (excluding Bangladesh, due to incomplete information) and 801 responses (743, excluding Bangladesh) received from final-year pharmacy students in the nine pilot countries. The collated dataset included 791 valid and complete responses, which met the sample size requirement (*N *= 783) to achieve adequate power (0.8) to detect small-effect size (*r *= 0.1) differences between groups.

**Table 1:** Sample characteristics

	**Sample** **(N = 791)**			**Gender** **(N = 783)**				**Age** **(N = 784)**	**Intention to migrate within next 5 years** **(N = 786)**					
	
Countries	N sampled (N = 974)*	N respondents (N = 791)	% of dataset	Female		Male		Mean	No		Short-term migration		Long term migration	
						
				N (478)	Sample %	N (305)	Sample %		N (373)	Sample %	N (159)	Sample %	N (254)	Sample %
Australia	405	334	42.2	222	67.3	108	32.7	22.2	157	47.3	95	28.6	80	24.1

Bangladesh	N/A	58	7.3	14	24.1	44	75.9	22.8	6	10.5	15	26.3	36	63.2

Croatia	110	96	12.1	87	90.6	9	9.4	22.5	82	86.3	5	5.3	8	8.4

Egypt	117	95	12.0	31	33.0	63	67.0	20.7	19	20.2	13	13.8	62	66.0

Nepal	33	31	3.9	12	38.7	19	61.3	23.1	8	25.8	2	6.5	21	67.7

Portugal	118	55	7.0	35	64.8	19	35.2	23.3	32	58.2	13	23.6	10	18.2

Singapore	81	60	7.6	50	83.3	10	16.7	23.0	38	63.3	6	10.0	16	26.7

Slovenia	65	25	3.2	18	72.0	7	28.0	22.4	22	88.0	3	12.0	0	Nil

Zimbabwe	45	37	4.7	9	25.7	26	74.3	23.3	9	24.3	7	18.9	21	56.8

Sample means					61.0		39.0	22.3		47.5		20.2		32.3

*Excluding Bangladesh

The mean age of respondents was 22.3 years (*SD *2.4). Sixty-one per cent of the total sample was female and the proportion of female respondents in each country ranged from 24.1% in Bangladesh to 90.6% in Croatia. The proportion of female students in each country was similar to that reported by an IPSF international study with a comprehensive database of pharmacy students (with the exception of Egypt and Zimbabwe, where comparisons were unavailable) [40]. Thus, the sample was assumed to be representative of the final-year pharmacy students in the pilot countries.

The intention to migrate varied between countries, with 47.5% of respondents overall who had no intention to migrate, 20.2% who intended short-term migration and 32.3% who intended long-term migration. Thus, half the respondents overall indicated an intention to migrate within the next five years.

### Principal Components Analysis

PCA yielded three factors that explained 46.4% of total variance. Factor 1 described the attitude towards the professional environment and status in their home country (10 items, *α *= 0.82). Factor 2 described the perception of the opportunity to develop a career and resources abroad (4 items, *α *= 0.75). Factor 3 described the attitude towards the sociopolitical environment in their home country (5 items, *α *= 0.67).

**Figure 1:**
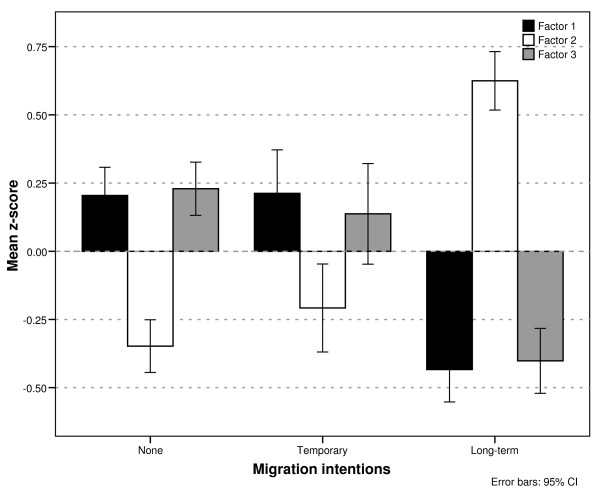
**Attitudes towards home country professional and sociopolitical environment and opportunities abroad by migration intention**.

There was a significant difference in all factors between respondents who did not intend to migrate compared to those who intended to migrate on a long-term basis, with more negative attitudes towards the home country environment and a more positive perception of opportunities abroad (Factor 1, *t*(579) = 7.9, *r *= 0.31, *p *< 0.001; Factor 2, *t*(543) = -12.8, *r *= 0.48, *p *< 0.001; Factor 3, *t*(601) = 8.2, *r *= 0.32, *p *< 0.001). It can be seen from Figure 1 that those students intending to migrate short-term had a similar profile of attitudes to those who did not plan to migrate (no significant difference) and had significantly different attitudes to students planning long-term migration (Factor 1, *t*(375) = 6.7, *r *= 0.33, *p *< 0.001; Factor 2, *t*(284) = -8.3, *r *= 0.44, *p *< 0.001; Factor 3, *t*(273) = 5.2, *r *= 0.30, *p *< 0.001).

### Two-step cluster analysis

Analysis revealed four case clusters that represented 86.7% of the dataset (13.3% were excluded, due to a missing value for one or more variables). Table 2 describes the significant defining characteristics of each cluster. In Table 2, the column describing attitudes towards the home country environment compares scores for both Factor 1 and 3; the column on attitudes towards opportunity abroad represents Factor 2. Each cluster is distinct in the intention to migrate, ranging from predominantly long-term migration intention (Cluster 1) to no intended migration (Cluster 4). Cluster 1 describes characteristics of a group that is most likely to migrate and Cluster 4 the least likely to migrate. Cluster 2 was associated with mostly long-term migration intentions. Cluster 3 describes a subpopulation that is most likely to migrate on a short-term basis.

**Table 2:** Cluster characteristics

**Clusters** **(N = 686)**	**Intention to migrate**	**Gender**	**Knowledge of other migrant pharmacist**	**Past pharmacy experience abroad**	**Attitudes towards home country environment**	**Attitudes towards opportunities abroad**
1 (N = 111)	Long-term intention	Mostly male	Yes	None	Strongly negative	Strongly positive

2 (N = 165)	Mostly long-term	Both	Mostly do not know	Yes	Negative	Positive

3 (N = 262)	Mostly short-term	Both	Mostly do know	None	Positive	Negative

4 (N = 148)	None	Female	No	None	Neutral	Neutral

## Discussion

Results showed a significant and medium-effect size difference in attitudes towards the professional and sociopolitical environment of the home country and perceptions of opportunities abroad between those who have no intention to migrate or short-term migration intentions and those who intend to migrate long-term. These attitudes, together with gender, knowledge of pharmacist migrant networks and past experiences abroad, are associated with an increased propensity for migration. The finding that attitudes towards the home environment and opportunities abroad may influence the intention to migrate supports previous findings that a broader set of both push and pull factors should be taken into consideration [10,16].

These identified factors provide a deeper understanding of the relationships between variables that influence the intention to migrate. However, factors relating to the country environment and context should not be assumed to be uniform across all respondents. Findings suggest that variance in attitudes is inherent within and between countries and thus cannot be assumed to be standardized.

The results also provide evidence to demonstrate that economic motivation for migration is not an independent, stand-alone factor in itself, but rather a component of a broader factor (as identified here as Factor 2) that takes into consideration the potential to develop both resources and a career abroad. This finding is a departure from previous studies of intention to migrate that all cite remuneration as a key independent influencing factor. This may be partly because their design prevented deeper analysis of relationships between factors [1,15,17,22-24].

Based on a broader framework of understanding derived from the results of this study, a number of inferences can be drawn relating to strategies to encourage retention. Such strategies should frame the issue of migration in context of the wider human resource agenda, thus viewing migration as a form of attrition or workforce exit (rather than a stand-alone phenomenon). To proceed from this rationale, countries experiencing a shortage of health workforce exacerbated by emigration, in addition to other forms of attrition such as change of profession, change to non-practising role, retirement and death, should prioritize interventions that encourage retention and enhance workforce and practitioner development.

Factor 1 (attitudes towards the professional status and practice environment towards the home country) refers to the need to improve working conditions and the professional interface with other health professionals and society. Planned interventions could employ non-financial incentives and human resource management tools, such as recognition by management, performance review and improving interprofessional working relationships, to uphold and strengthen the professional ethos of health professionals, a key determinant of motivation and retention [41].

Factor 2 (perceptions of the opportunity to develop resources and career prospects abroad) recognizes the influence of the labour market in creating demands and the linkage of issues relating to remuneration and professional development. This supports the rationale for workforce strategies to enhance retention through investment in professional development opportunities in terms of career progression pathways (professional role development) and training, despite the existence of relatively lower salaries compared with those offered abroad [3,41,42]. The results suggest that combined strategies addressing professional development opportunities as well as ensuring appropriate remuneration is warranted, rather than stand-alone efforts in either.

Factor 3 (attitudes towards the sociopolitical environment in the home country) indicates the influence of factors beyond the individual. It would be important to distinguish here between two sets of factors in the sociopolitical environment. One set relates to factors within the control of the health and labour sectors, such as health systems, policies and public and private sector dynamics. The other set of factors relates to those likely beyond the scope of the health and labour sectors, yet play a significant role in influencing the intention to migrate, such as political stability, human rights (including the right to own and exchange property and the right to operate a business without undue political interference), rule of law (enforced by an independent judiciary), free speech, cultural issues and social development [16].

The negative attitude towards the professional and sociopolitical environment and positive perception of opportunities abroad were associated with the intention to migrate, particularly on a long-term basis (Table 2). Those intending long-term migration may be a subpopulation of the workforce that will be difficult to retain or encourage to return from abroad. However, there appears to be an opportunity for maximized benefits from migration with those who intend to migrate on a short-term basis, as described by Cluster 3. Results suggest that the intention to migrate should be defined as short-term or long-term in nature, rather than pooled.

Short-term migration intentions are clustered with defining characteristics that are essentially different from those of long-term migration intentions. Those planning short-term migration are more positive towards their home country and more negative towards opportunities abroad. Exploration of the potential for return migration in those who intend short-term migration was not within the scope of this study. However, this will be explored in a follow-up study by examining scenarios in which return migration is more likely. Further study is warranted to build on the limited existing evidence base for understanding return migration and the distinction in characteristics between long-term and short-term migration intentions [1,2].

Gender plays an important role; it is clear that long-term migration is selective towards males. Cluster 4 describes a subpopulation within the sample that is entirely female with no intention to migrate, unaware of other pharmacists who have migrated and hold ambivalent attitudes. By contrast, Cluster 1 describes a subpopulation that is mostly male, has access to migrant pharmacist networks and holds strong negative attitudes towards their home country and strong positive attitudes towards opportunities abroad. Neither cluster has had any past pharmacy experience abroad. Further research is planned to better understand the gender dynamics. Some evidence that migration is selective towards males exists, though this depends on the country context and demographics within the profession [23].

Knowledge of a pharmacist who has migrated abroad also plays a significant role and alludes to the potential migration network effect (Table 2). This tends to facilitate migration by reducing the associated costs and risks of migration and increasing the potential gains [1,9,43]. This is closely associated with the intention to migrate, though the direction of causation is unclear.

Those who intend to migrate on a long-term basis tend to know of a migrant pharmacist, while those who do not intend to migrate do not. This could be explained in different ways, depending on the direction of causality. Those who intend to migrate on a long-term basis may actively seek out migrant pharmacists. Or, prior knowledge of a pharmacist who has migrated may determine the choice of training (in this case, pharmacy education) and influence the intention to migrate on a long-term basis.

Should a set of societal values, expectations and perceived behavioural norms relating to an established migration flow exist (also referred to as "culture of migration") in specific country contexts, as was found to be the case in physician migration from Ghana, it is possible that prior knowledge of a migrant pharmacist potentially influences the intention to migrate [3]. The converse may be the case in countries without an established culture of migration.

Research and policy debates on the migration of health professionals tend to centre on "push-pull" theories, supportive of mainstream oversimplification of a complex phenomenon. There is a paucity of research on factors influencing migration and potential opportunities for policy intervention to strengthen human resources and health systems in countries, particularly concerning the pharmacy workforce.

A multidimensional understanding of factors influencing the intention to migrate, taking into account the relationships between variables, is proposed. Further research is required to build a theoretical framework that encompasses this approach.

The authors are in the process of analysing the results of the next round of this international study (13 countries), which aims to further explore the complex dynamics and relationships between factors, gender, countries of intended migration, linguistic and migrant network ties and return migration. The country-specific policy context will also be examined to explore the association between attitudes of practitioners, the policy environment and policy options to strengthen the workforce.

## Conclusion

There is a significant difference in attitudes towards the professional and sociopolitical environment of the home country and perceptions of opportunities abroad between those who have no intention to migrate and those who intend to migrate on a long-term basis. Attitudes of students planning short-term migration were not significantly different from those of students who did not intend to migrate. These attitudes, together with gender, knowledge of migrant networks and past experiences abroad, are associated with an increased propensity to migrate. The economic motivation for migration is not an independent factor in itself. This research, together with other emerging evidence and policy papers, suggests that the migration of health professionals is neither the cause nor the solution to the human resource for health crisis [7,8]. Given that the country context is crucial in determining these attitudes and thus migration intentions, research and policy should approach migration as a form of workforce attrition, rather than as a stand-alone phenomenon, and view migration as a symptom of other root causes.

## Competing interests

The authors declare that they have no competing interests.

## Authors' contributions

TW, SC and IB jointly formulated the study design, obtained and analysed the data, interpreted the findings and wrote the article. All authors had access to all data in the study and had final responsibility for the decision to submit this manuscript for publication.
